# Can laptops be left inside passenger bags if motion imaging is used in X-ray security screening?

**DOI:** 10.3389/fnhum.2013.00654

**Published:** 2013-10-18

**Authors:** Marcia Mendes, Adrian Schwaninger, Stefan Michel

**Affiliations:** ^1^School of Applied Psychology, University of Applied Sciences and Arts Northwestern Switzerland (FHNW)Olten, Switzerland; ^2^Center for Adaptive Security Research and Applications (CASRA)Zürich, Switzerland

**Keywords:** aviation security, X-ray screening, threat detection, human factors, motion imaging, multiple views, laptop screening

## Abstract

This paper describes a study where a new X-ray machine for security screening featuring motion imaging (i.e., 5 views of a bag are shown as an image sequence) was evaluated and compared to single view imaging available on conventional X-ray screening systems. More specifically, it was investigated whether with this new technology X-ray screening of passenger bags could be enhanced to such an extent that laptops could be left inside passenger bags, without causing a significant impairment in threat detection performance. An X-ray image interpretation test was created in four different versions, manipulating the factors packing condition (laptop and bag separate vs. laptop in bag) and display condition (single vs. motion imaging). There was a highly significant and large main effect of packing condition. When laptops and bags were screened separately, threat item detection was substantially higher. For display condition, a medium effect was observed. Detection could be slightly enhanced through the application of motion imaging. There was no interaction between display and packing condition, implying that the high negative effect of leaving laptops in passenger bags could not be fully compensated by motion imaging. Additional analyses were carried out to examine effects depending on different threat categories (guns, improvised explosive devices, knives, others), the placement of the threat items (in bag vs. in laptop) and viewpoint (easy vs. difficult view). In summary, although motion imaging provides an enhancement, it is not strong enough to allow leaving laptops in bags for security screening.

## Introduction

A secure air transportation system is vital for society and economy. Aviation security measures have been increased substantially in response to several successful and attempted terrorist attacks since September 11, 2001. One major aspect in this field is the mandatory process of baggage screening using X-ray machines. Before entering the secure area of an airport, all passengers, as well as members of airline and airport staff have to pass the security checkpoints to have themselves and all their belongings screened. The security checkpoint is a socio-technical system consisting of human and technical elements, working together. The goal is that no threat items are brought past security checkpoints and onto an airplane. Strong efforts are being made in order to improve and further develop X-ray screening equipment. Yet, the final decision whether threat items are contained in the baggage still relies on human operators (screening officers) who visually inspect the X-ray images provided by the machine. As a consequence, man-machine system performance depends on human factors and display technology (e.g., Bolfing et al., 2008; Koller et al., 2008; von Bastian et al., 2008, 2010; Michel and Schwaninger, 2009; Graves et al., 2011). When evaluating new technological developments with regard to their added value for security screening purposes, this should be taken into account appropriately (see also Yoo and Choi, 2006; Yoo, 2009).

In X-ray screening, three image-based factors have been identified as relevant for human operators to detect threat items in X-ray images (Schwaninger, 2003b; Hardmeier et al., 2005, 2006; Schwaninger et al., 2005a). The first one is the view difficulty of an object, resulting from the position of a threat item in a bag (effect of viewpoint). The second factor is the superposition of an item by other objects contained in the bag (effect of superposition). The third factor refers to the complexity of a bag, which depends on the number and type of objects in the bag (effect of bag complexity). The intensity with which X-rays can penetrate through materials in a bag depends on the specific material density of a substance (e.g., Brown et al., 1995). Therefore, the material density of the items contained in a bag will also affect the factors superposition and bag complexity and thus will influence the difficulty to detect threat items. Schwaninger et al. (2005b) have developed algorithms to automatically estimate X-ray image difficulty based on viewpoint, superposition, and bag complexity. Their algorithms were highly correlated with human perception of the above mentioned image-based factors and could well predict human threat detection performance (see also Schwaninger et al., 2007; Bolfing et al., 2008).

State-of-the-art X-ray screening equipment is able to provide high quality images with good image resolution. Yet, the detection of threat items in X-ray images remains a challenging task for screening officers and becomes even more difficult when dense objects, such as large electronic devices, are contained in the baggage. Due to their compact construction, electronic devices (e.g., laptops) are hard to penetrate. Hence, they can conceal other parts of luggage or could be used to intentionally hide threat items (e.g., an improvised explosive device, IED). Especially when single view X-ray systems are used or even multi-view systems, if the additional views do not provide enough meaningful information, the inspection becomes difficult. Threat items which are behind, in front of, or hidden inside a laptop case become very challenging or even impossible for human operators to recognize (see also von Bastian et al., 2008). In a previous paper, Mendes et al. (2012) documented how threat detection can be substantially impaired when laptops are not taken out of passenger bags and a threat item (e.g., an IED) is placed either behind, in front of, or within a laptop. The present paper extends these results by investigating how a new technology which allows presenting bags in multiple views as an image sequence (i.e., motion imaging) could possibly reduce such an impairment.

Considering the large number of views which can be produced by a single object, the question arises how objects can be recognized when presented in unusual views. In the object recognition literature, two types of theories can be distinguished (see Peissig and Tarr, 2007; Kravitz et al., 2008): viewpoint-invariant theories (e.g., Marr, 1982; Biederman, 1987) and viewpoint-dependent theories (e.g., Poggio and Edelman, 1990; Bülthoff and Edelman, 1992; Tarr, 1995). Most viewpoint-invariant theories assume that objects are stored in visual memory by their component parts and their spatial relationship (see Marr and Nishihara, 1978; Biederman, 1987). Once a particular object has been stored, recognition of that object should be unaffected by the viewpoint (including novel viewpoints), given that the necessary features can be recovered from this view (Burgund and Marsolek, 2000). The viewpoint-dependent theories propose that objects are not stored in memory as rotation invariant structural descriptions, but in a viewer centered format. Thus, if an object has never been seen from a certain viewpoint and is therefore not stored in visual memory, recognition is impaired if view-invariant features are not available (Kosslyn, 1994; Bülthoff and Bülthoff, 2006; Schwaninger, 2005). Several studies on viewpoint-dependent theories could show that viewpoint can strongly affect recognition performance (e.g., Bülthoff and Edelman, 1992; Edelman and Bülthoff, 1992; Humphrey and Khan, 1992; Graf et al., 2002). Even though our visual perception can be considered highly robust with respect to changes of viewpoint, we are more facile with certain views relative to others, such as often encountered views and views that make larger numbers of surfaces available (Palmer et al., 1981; Blanz et al., 1999). Such views have also been referred to as “canonical” views. Research in aviation security X-ray screening has shown that threat items are easier to identify when depicted in frontal (canonical) views than when horizontally or vertically rotated (e.g., Michel et al., 2007; Bolfing et al., 2008; Koller et al., 2008). Consequently, having machines featuring multiple X-ray images of the same bag from different viewpoints could ease recognition of threat items in passenger bags for screening officers.

At present, most of the machines deployed at airports provide single view images, which do not allow screening officers to analyze an image from different viewpoints. A human operator will only be able to identify a threat item and make a correct decision if the threat can be recognized in the provided single view image (Schwaninger, 2003b; Schwaninger et al., 2005a; Graves et al., 2011). Considering the above mentioned image based factors (viewpoint, superposition and bag complexity) and the density of electronic devices, it becomes evident why most international and national regulations specify that portable computers and other large electronic devices shall be removed from passenger bags and screened separately at security checkpoints (e.g., the current regulation of the European Comission, 2010). Based on the model by Schwaninger et al. (2005b) one would predict that leaving laptops in passenger bags results in decreases of threat detection performance due to increases of superposition and bag complexity. Threat items placed behind, in front of, or inside a laptop could become very challenging for human operators to detect. Moreover, recognition would become additionally challenging if in the provided X-ray image the threat item would be depicted from a difficult viewpoint (e.g., vertically or horizontally rotated).

**Figure 1:**
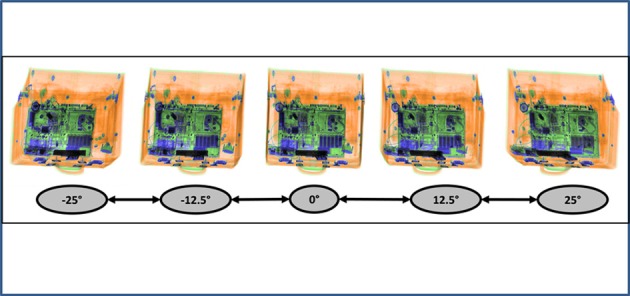
**Example of motion imaging X-ray images provided by the machine evaluated in this study**. The image in the middle shows the initial image (0°).

This study was conducted to examine the above mentioned effects by comparing conventional single view display technology to a new technology. More specifically, a new X-ray screening machine featuring “motion imaging” was tested. “Motion imaging” means that five images are available, which are rotated around the vertical axis. These can be either displayed in a short video sequence or can each be statically viewed. In relation to the initial image (0°), the angles of the five images are −25°, −12.5°, 0°, 12.5°, 25° (see Figure 1).

One could hypothesize that through the application of motion imaging and the availability of multiple views, recognition of certain objects could become easier. There are several possible advantages dynamic displays may confer over static ones (Vuong and Tarr, 2004). For example, object motion may enhance the recovery of information about shape (e.g., Ullmann, 1979). Furthermore, it may provide observers with additional views of objects (Pike et al., 1997), or it may allow observers to anticipate views of objects (Mitsumatsu and Yokosawa, 2003). Moreover, when objects rotate in depth, certain features can become visible while others become obscured (Vuong and Tarr, 2004). Thus, objects could become less superimposed and could possibly be displayed from an easier viewpoint (i.e., from a more canonical perspective).

The first goal of our study was to determine whether motion imaging improves detection of threat items in passenger bags. The second goal was to investigate whether leaving laptops in passenger bags results in a decrease of detection performance (effect of superposition and bag complexity), while the third goal was to evaluate whether such an effect can be compensated when motion imaging is available. Additional analyses were carried out to examine effects depending on different threat categories (guns, IEDs, knives, others), the placement of the threat items (in bag vs. in laptop) and the viewpoint effect (easy vs. difficult view).

## Methods and materials

An image interpretation test containing bags and laptops was created in four versions to examine the factors display condition (single vs. motion imaging) and packing condition (laptops inside vs. laptops outside). Each test version differed with regard to these two factors (see section Experimental Design). Four experimental groups with certified screening officers were formed. Each group conducted one of the test versions. Detection performance scores and reaction times (RTs) of all groups were compared to evaluate the effects of the above mentioned factors.

### Participants

**Figure 2:**
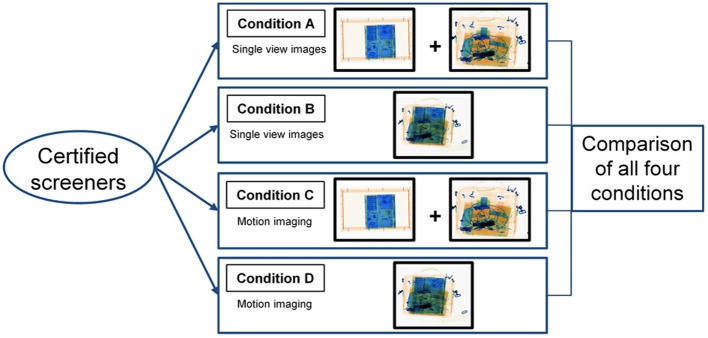
**Experimental design (between-participants) for the comparison of the four different conditions**.

The study was conducted with 80 airport security screening officers employed at an international European airport. All participants were certified screeners, meaning they were all qualified, trained and certified according to the standards set by the national appropriate authority (civil aviation administration) and consistent with the European Regulation (European Comission, 2010). The screening officers were randomly distributed into four different experimental groups (A, B, C, and D, 20 per group). Figure 2 illustrates the experimental design. In order to verify that all experimental groups were comparable with regard to the screeners' X-ray image interpretation competency, all participants conducted the X-Ray Competency Assessment Test (X-Ray CAT) before the main experiment was carried out. The X-Ray CAT for cabin baggage screening is a standardized instrument to measure X-ray image interpretation competency of airport security screening officers and has been applied in several previous scientific studies (Koller and Schwaninger, 2006; Michel et al., 2007; Koller et al., 2008). It is currently used for screener certification at several European airports. The test consists of 256 trials and is based on 128 different color X-ray images of passenger bags, which are each used twice: once without (non-threat image) and once containing a threat object (threat image). For more information on the X-Ray CAT see Koller and Schwaninger (2006). Average detection performance scores (A′)^1^ of all four groups were compared using *post-hoc* pairwise comparisons with Bonferroni correction. No significant differences between the groups could be found (all *p* values >0.05), implying that they were comparable regarding their image interpretation competency. The average age of the participants was *M* = 40.69 years (*SD* = 10.78), with a range between 22 and 58 years. 53% of the participants were female. The average amount of job experience was *M* = 4.95 years (*SD* = 4.49, range: 0.5–23 years). Between-participants analyses of variance showed no differences between the experimental groups with regard to age [*F*_(3, 76)_ = 1.57, *p* = 0.204, η^2^ = 0.058] or job experience [*F*_(3, 74)_ = 0.66, *p* = 0.579, η^2^ = 0.026].

### Experimental design

All experimental groups conducted a computer-based X-ray image interpretation test. During the test, color X-ray images of passenger bags and laptops were displayed, sometimes containing threats (threat images) and sometimes without any threat items (non-threat images). Images were displayed in random order. All participants were exposed to every image and had to decide whether the bags and laptops could be regarded as harmless (OK) or whether they contained a threat item (NOT OK). Each test condition differed with regard to the factors display condition (single view vs. motion imaging) and packing condition (laptops inside vs. outside of passenger bags). Figure 2 displays the experimental design of the study. The following four different experimental conditions were conducted and compared to examine the effects and interactions of the above mentioned two factors using a between-participants design:

- Single view images, laptops and passenger bags screened separately.
- Single view images, laptops are left inside the passenger bags.
- Motion imaging, laptops and passenger bags screened separately.
- Motion imaging, laptops are left inside the passenger bags.

In all test conditions the same bags were presented to the screening officers. Originally, every bag contained a laptop. In conditions A and C the laptops were taken out of the bag and screened separately, whereas in conditions B and D the laptops were left inside the passenger bags. This allowed examining the effects of superposition and bag complexity caused by laptops. Figure 2 illustrates the two different packing conditions (laptop inside vs. laptop outside).

In conditions A and B, images of the baggage and laptop could only be seen from one single viewpoint. Conditions C and D allowed examining the images from different viewpoints through motion imaging. As explained in the introduction, one important objective of this study was to test whether motion imaging could enhance the inspection of passenger bags to such an extent that laptops could be left inside passenger bags without affecting detection performance negatively. Would detection performance scores still be significantly higher in condition A compared to D, one could conclude that the detection of threat items is significantly impaired when laptops are left inside passenger bags, even when motion imaging is available.

### Image interpretation test

**Figure 3:**
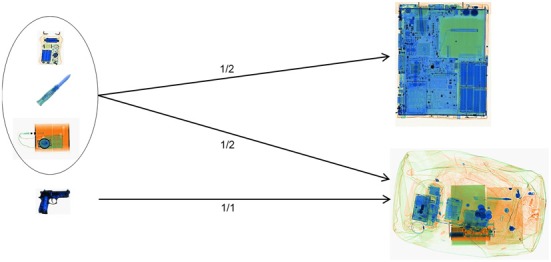
**Placement of the threat items**.

The image interpretation test was based on a representative set^2^ of 96 passenger bags (defined by screening experts from a specialized police organization), all of which originally contained laptops. All test images were recorded with the machine evaluated in this study. The test images were created and recorded in collaboration with aviation security experts from a specialized police organization and former airport security screening officers now employed by CASRA. As explained above, in conditions A and C the laptops were taken out of the bags and recorded separately, whereas in conditions B and D the laptops were left inside the bags. Each bag/laptop-combination was used twice, once containing a threat item in either the bag or the laptop, and once without any threat item. The test contained a representative sample of threat items selected and developed (the IEDs in laptops) by experts from an airport police department. These could be divided into four different threat categories: guns, IEDs, knives and other threat items (e.g., electric shock devices, etc.). For all categories except guns, in half of the cases the threat items were placed in the bag, while in the other half of the cases the threat items were placed within the laptop (see Figure 3). Due to their size, it would not have been realistic to place guns inside a laptop. Moreover, the factor viewpoint was included in the test design. For those threat items placed inside the bags, half were positioned in easy views and half in difficult views. Easy view means that threat items were depicted from a frontal/canonical view in the X-ray image, while for difficult view the threat items were horizontally or vertically rotated. All the threat items placed inside the laptop cases were positioned in easy views. As laptops are comparably flat, it would have been difficult to place threat items in vertically or horizontally rotated positions. The IEDs which were placed inside the laptops were specifically built into the cases. It must be considered that since an IED consists of several component parts, it becomes more difficult to determine what the canonical view and thus an easy view would be. Each threat category contained 24 items. Therefore, the number of test images for the conditions were the following:

- **Tests A and C (laptops and bags screened separately):**
  4 × 24 threat images (60 bags and 36 laptops)
  +96 non threat *BAG* images
  +96 non threat *LAPTOP* images
  = *288 test images*
- **Tests B and D (laptops inside bags):**
  4 × 24 combined threat images (60 threats in bags and 36 threats in laptops)
  +96 combined non threat images
  = *192 test images*

### Procedure

All participants were invited to the experimenters' facilities to conduct the test. Four computer workstations with the corresponding consoles of the tested machine and 19′ TFT monitors were set up in a normally lit room. X-ray images covered about 2/3 of the computer screen. The distance to the monitor was ~60 cm. Four participants at a time were tested. Before the test started, all participants received a short introduction by the test supervisor, explaining the test procedure and introducing the new technology of motion imaging. All participants were able to try out the console and view test images for ~20 min, in order to become familiar with the images, the technology and the handling of the console. Pre-testing had shown that this amount of time was enough to get well acquainted with the console and it was also recommended by the manufacturer. After a break of 10 min the actual test started. Tests were conducted quietly and individually, and under supervision. The test images remained on the screen until the participant either pressed the “OK” or “NOT OK” and the “move belt forward” button. RTs were measured in milliseconds and correspond to the amount of time it took for a screening officer to come to a decision and press the “OK” or “NOT OK” button after the first image pixel of the bag/laptop appeared on the screen. There was no time limit set for viewing an image. However, participants were instructed to inspect the images as quickly and accurately as possible. Breaks of 10 min were taken in 30 minute-cycles, to avoid eyestrain and fatigue, and to make sure that especially those participants conducting tests A and C (288 images instead of 192 images, see section Image Interpretation Test) would not become too tired toward the end. All participants completed the test in less than 2 h, including breaks.

## Results and discussion

According to signal detection theory (Green and Swets, 1966), there are four possible outcomes to a screener's response when judging an X-ray image as either OK or NOT OK: hit, false-alarm, correct rejection and miss (Schwaninger, 2003a; Hofer and Schwaninger, 2004). In this study, A′ was applied as a measure for detection performance (Pollack and Norman, 1964). A′ is a measure of sensitivity which is commonly used for a variety of tasks including screener certification and competency assessments (Hofer and Schwaninger, 2004; Koller and Schwaninger, 2006; Michel et al., 2010). It considers the hit rate as well as the false-alarm rate and can be calculated using the following formula (Grier, 1971):

(1) 0.5+[(H−F)(1+H−F)]/[4H(1−F)]

(2) 0.5+[(F−H)(1+F−H)]/[4F(1−H)]

*H* is the hit rate and *F* the false alarm rate. If performance is below chance, i.e., when *H* < *F*, equation (2) must be used (Aaronson and Watts, 1987).

Due to the security confidential nature of performance values, these are not displayed in this paper. In order to provide meaningful results, relative differences and effect sizes are reported. All reported effect sizes are interpreted based on Cohen (1988). For *t*-tests, *d* between 0.20 and 0.49 represents a small effect size; *d* between 0.50 and 0.79 represents a medium effect size; *d* ≥ 0. 80 represent a large effect size. For analysis of variance (ANOVA) statistics, η^2^ between 0.01 and 0.05 represents a small effect size; η ^2^ between 0.06 and 0.13 represents a medium effect size; η^2^ ≥ 0.14 represents a large effect size.

### Comparison of detection performance by condition

**Figure 4:**
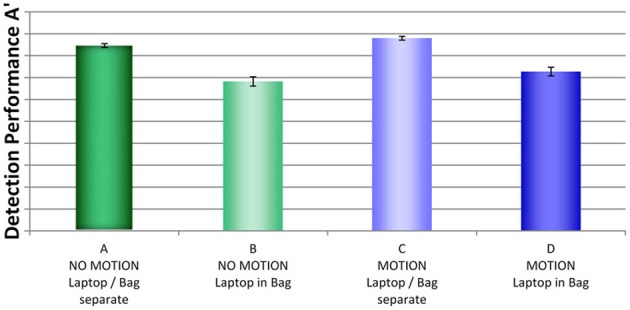
**Mean detection performance A' with standard errors of the mean for all four conditions (A–D)**. For security reasons, actual A' performance scores are not reported.

Figure 4 shows a comparison of detection performance scores by condition (A, B, C, and D)^3^. Most remarkable seems to be the effect of packing condition. Performance was much better in conditions A and C, where laptops and bags were screened separately, compared to conditions B and D, where laptops were left inside the passenger bags. The graph also suggests that performance was slightly better when motion imaging was available (condition C compared to A and condition D compared to C, respectively). The ANOVA with the between-participants factors display condition (no motion vs. motion) and packing condition (laptop separate vs. laptop in bag) revealed a large main effect for packing condition, *F*_(1, 76)_ = 105.22, *p* < 0.001, η^2^ = 2.0, and a medium main effect for display condition, *F*_(1, 76)_ = 5.05, *p* < 0.05, η^2^ = 2.0. There was no interaction between display and packing condition, *F*_(1, 76)_ = 0.361, *p* = 0.55, η^2^ = 2.0. Thus, although motion imaging enhanced detection performance slightly, it could not compensate the negative effects on detection performance resulting from leaving laptops inside bags. Further, the direct comparison of condition D (motion imaging available, laptops in bags) and condition A (no motion imaging available, laptops and bags screened separately) revealed a highly significant effect, *t*_(26)_ = 5.89, *p* < 0.001, *d* = 1.86. This also shows that although motion imaging did improve detection performance (as shown by the main effect in the ANOVA), the large negative effect of packing condition could not be compensated.

In sum, the results imply that the packing condition had a high impact on detection performance. Motion imaging resulted in better detection but could not fully compensate the effect of packing condition (i.e., impaired detection when leaving laptops in passenger bags). The large main effect for packing condition is consistent with the assumption that the well-documented effects of superposition and bag complexity (Schwaninger et al., 2005a,b, 2007; Hardmeier et al., 2005, 2006; Bolfing et al., 2008; von Bastian et al., 2008) increase when laptops are left in passenger bags, resulting in impairments of threat detection performance.

**Figure 5:**
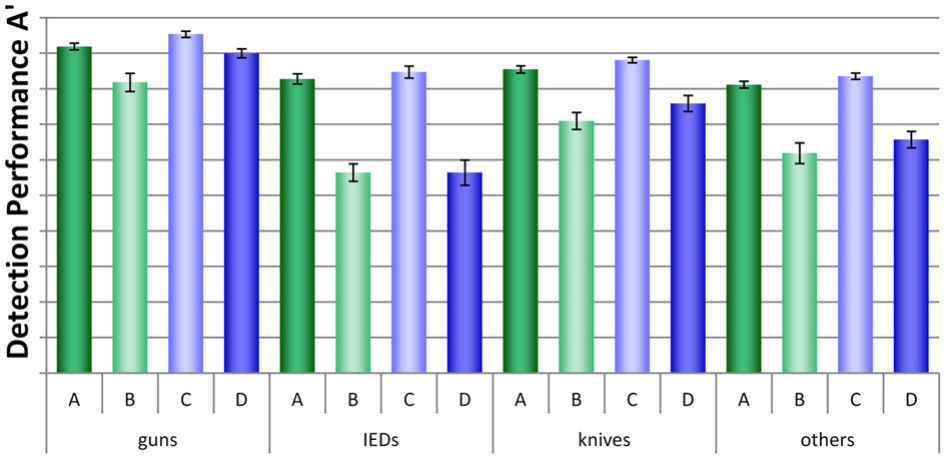
**Mean detection performance A' with standard errors of the mean for all four conditions (A–D) and each threat category (guns, IEDs, knives, others)**.

A more detailed analysis was conducted by looking at each threat category separately. As can be seen in Figure 5, large differences between conditions, but also between threat categories were found. A mixed-design ANOVA with the within-participants factor threat category (guns, IEDs, knives, others) and the between-participants factors display condition (no motion vs. motion) and packing condition (laptop separate vs. laptop in bag) revealed large significant main effects for the factors threat category and packing condition and a medium effect for display condition (for details, see Table 1A). The interaction between threat category and packing condition was also highly significant, implying that leaving laptops in passenger bags affected performance differently, depending on threat category. None of the other interactions reached statistical significance. As Figure 5 indicates, IEDs and other threats were most difficult to detect, especially in conditions B and D. In general, a slight advantage of motion imaging could be observed (compare condition C to A, and D to B), which according to Figure 5 was most evident for guns.

**Table 1:** **Results of the ANOVAs conducted with detection performance (A′) as dependent variable^4^**.

**Detection performance**	***df***	***F***	**η^2^**	***p*-value**
**(A)**				
Threat category (*T*)	2.04, 155	186.96	0.711	<0.001
Display condition (*D*)	1, 76	4.49	0.056	<0.05
Packing condition (*P*)	1, 76	108.12	0.587	<0.001
*T* × *D*	2.04, 155	2.43	0.031	0.09
*T* × *P*	2.04, 155	42.21	0.357	<0.001
*D* × *P*	1, 76	0.25	0.003	0.62
*T* × *D* × *P*	2.04, 155	1.15	0.015	0.320
**(B)**				
View difficulty (*V*)	1, 76	14.23	0.158	<0.001
Threat category (*T*)	2.23, 170	122.89	0.618	<0.001
Condition (*C*)	3, 76	28.78	0.532	<0.001
*V* × *C*	3, 76	0.33	0.013	0.805
*T* × *C*	6.70, 170	5.07	0.167	<0.001
*V* × *T*	2.38, 181	52.03	0.406	<0.001
*V* × *T* × *C*	7.12, 181	2.66	0.095	<0.05

Additionally, we conducted direct comparisons between conditions D (motion imaging available, laptops in bags) and A (no motion imaging available, laptops and bags screened separately) for each threat category, to further examine whether for certain threat types the negative effect on detection performance of leaving laptops in bags could be fully compensated by motion imaging. For all threat categories except guns, large significant differences were revealed (see Table 2). This further indicates that even though motion imaging did improve detection performance (as shown by the main effect in the ANOVA, see above), it could not compensate the large negative effect of packing condition. Only for the detection of guns, motion imaging seemed to have helped to compensate the negative effect of leaving laptops in bags (which could explain the marginally significant interaction (*p* = 0.09) between threat category and display condition in Table 1A).

**Table 2:** **Results of the two-tailed independent samples *t*-tests comparing detection performance A′ between conditions A and D for each threat category (guns, knives, IEDs, others)^5^**.

	***t*_(35)_**	***t*_(25)_**	***t*_(27)_**	***p***	***d***
A–D (guns)	1.22			0.23	0.39
A–D (IEDs)		6.90		<0.001	2.37
A–D (knives)			3.85	<0.01	1.30
A–D (others)		6.13		<0.001	2.10

**Figure 6:**
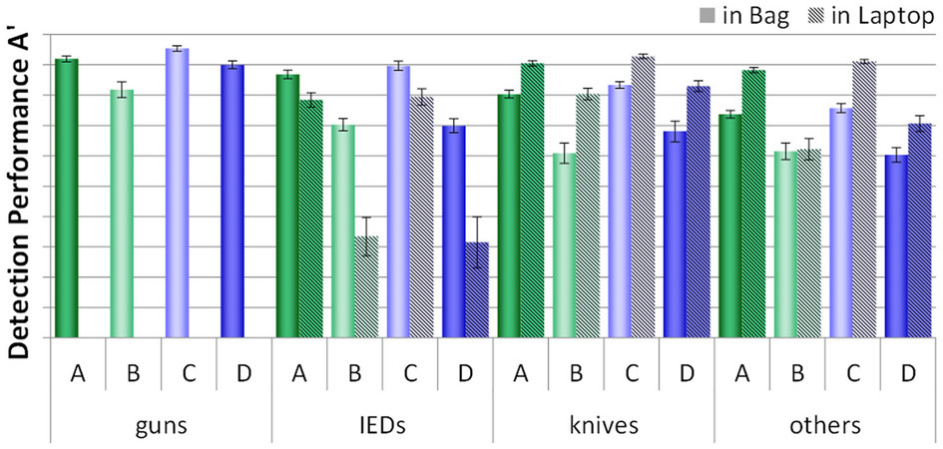
**Mean detection performance A' with standard errors of the mean for all four conditions (A–D) with regard to the placement of a threat item (in bag vs. in laptop) for each threat category (guns, IEDs, knives, others)**.

As described earlier, half of the threat items were placed inside laptops and half were placed inside the bags (except for guns, which could not be place inside laptops). Figure 6 displays how detection performance differed for each condition with regard to threat category and the placement of threat items. Again, threat items were detected better when the bags and laptops were screened separately (conditions A and C). Planned comparisons were conducted for each condition and threat category (except for guns, as all guns were placed inside the bags), to compare the differences between detection performance with regard to the placement of threats for each condition (see Table 3). Biggest differences were found for IEDs. For each condition, detection performance was worse when the IEDs were built into the laptops, compared to when they were placed inside the bags. However, while for conditions A and C detection performance was still relatively high, the scores achieved in conditions B and D were much lower for the IEDs within the laptops. For the threat categories knives and others, in most conditions detection performance was higher when these were placed inside the laptops. This could be explained by the fact that all threat items placed within the laptops were positioned in easy views (see Method section), and thus were easier to recognize. For IEDs, this effect was not observed. As the IEDs were specifically built into the laptops and since an IED consists of several component parts, it becomes more difficult to determine what actually the canonical/frontal view and thus an easy view would be (see Method section).

**Table 3:** **Results of two-tailed paired samples *t*-tests comparing the detection performance A' with regard to the placement of each threat item (in laptop vs. in bag) for each threat category (guns, IEDs, knives, others) and each condition (A–D)**.

	***t*_(19)_**	***p***	***d***
**IEDs**			
A	3.30	<0.01	0.74
B	5.98	<0.001	1.34
C	3.76	<0.01	0.84
D	4.83	<0.001	1.08
**KNIVES**			
A	−12.68	<0.001	−2.84
B	−7.76	<0.001	−1.73
C	−9.07	<0.001	−2.03
D	−5.32	<0.001	−1.19
**OTHERS**			
A	−14.24	<0.001	−3.18
B	−0.31	0.76	−0.07
C	−11.23	<0.001	−2.51
D	−5.45	<0.001	−1.22

**Figure 7:**
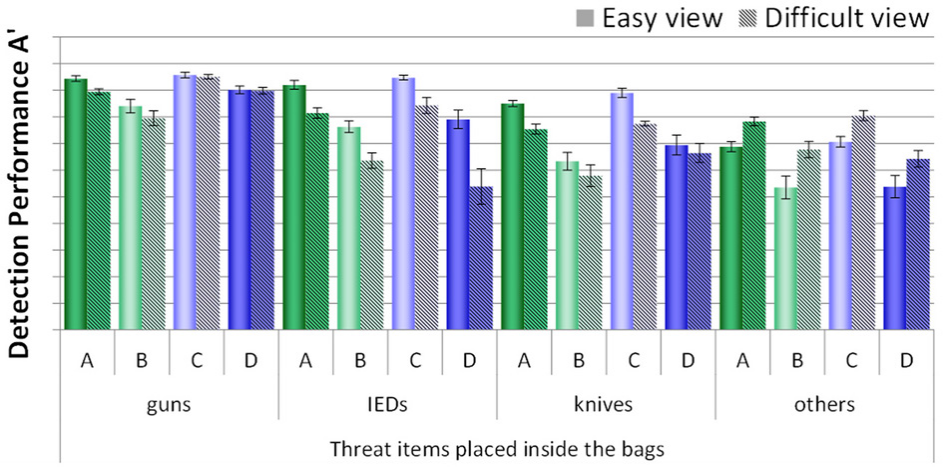
**Mean detection performance A' with standard errors of the mean for all four conditions (A–D) with regard to the view difficulty (easy vs. difficult view) for each threat category (guns, IEDs, knives, others)**. Only threat items which were placed inside the bags are included.

In order to examine the viewpoint effect and whether this effect was influenced by condition, detection performance scores of all conditions were compared, broken up by easy vs. difficult view (see Figure 7). Since all threat items placed inside the laptop cases were positioned in easy views, this analysis was only conducted for the threat items placed inside the bags. A mixed-design ANOVA with the within-participants factors view difficulty (easy vs. difficult view) and threat category (guns, IEDs, knives, others) and the between-participants factor condition (A, B, C, D) revealed large significant main effects for all three factors (see Table 1B). There was no significant interaction between view difficulty and condition, while all other interactions were significant. Therefore, a viewpoint effect could clearly be observed, which differed with regard to threat category. However, view difficulty was not significantly affected by condition. Interestingly, as Figure 7 indicates, throughout all conditions guns, IEDs and knives were detected better when depicted in easy views, while for the category others this was the other way around. The category others contained a very heterogeneous group of threat items (e.g., pepper spray, taser, throwing star, etc.). Hence, it could have been that the screening officers were more familiar with those threat items positioned in difficult views, and therefore recognized these more easily. As Figure 7 further indicates, for the category guns, motion imaging seemed to have been of help to reduce the viewpoint effect (see conditions C and D). This is consistent with the results reported above (see Table 2) and makes sense if one takes into account that guns change their shape more drastically than other objects when rotated. Thus, motion imaging can be more effective for supporting the recognition of guns.

### Comparison of reaction times by condition

**Figure 8:**
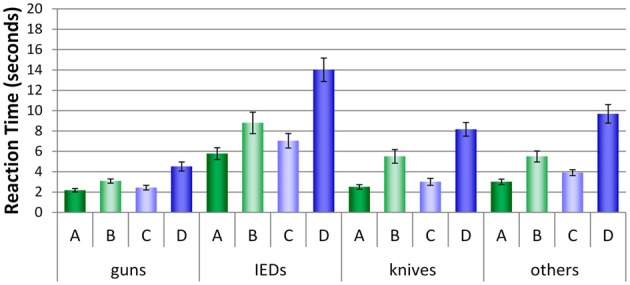
**Mean reaction times (s) with standard errors of the mean for all four conditions (A–D) broken up by threat categories (guns, knives, IEDs, others)**.

Figure 8 shows the average reaction times (RTs, converted into seconds) for all conditions and threat categories. For all categories, a similar pattern can be observed: More time was needed in conditions B and D where laptops were left inside the bags. Most time was needed in condition D, where motion imaging was available. As Figure 8 implies, remarkable differences can be observed between the threat categories and conditions. A repeated-measures ANOVA (see Table 4) revealed large significant main effects for the factors threat category (guns, IEDs, knives, others) and condition (A, B, C, D). The interaction between both factors was also significant, implying that the size of the differences in RTs between the conditions varied with regard to threat category. As displayed in Figure 8, all conditions achieved fastest RTs for the category guns, while longest RTs are clearly observed for the category IEDs.

**Table 4:** **Results of the repeated-measures ANOVA conducted with reaction time (RT)**.

**Reaction time**	***df***	***F***	**η^2^**	***p*-value**
Threat category (*T*)	1.89, 144	155.04	0.671	<0.001
Condition (*C*)	3, 76	25.26	0.499	<0.001
*T* × *C*	5.67, 144	8.11	0.242	<0.001

**Figure 9:**
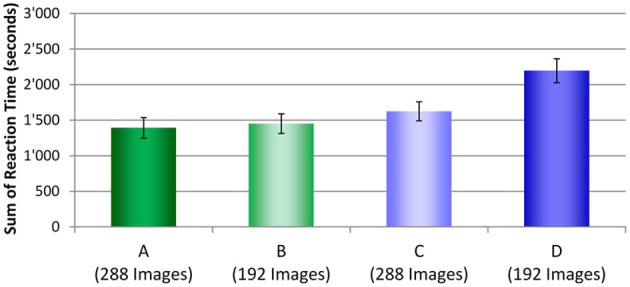
**Sum of reaction times (s) averaged across participants with standard errors of the mean for all four conditions (A–D)**.

To determine which condition actually took the longest time to complete the test all RTs for each security screener in each condition were summed and averaged across screening officers. Figure 9 displays these results. As described in the Method section, for test conditions A and C where laptops and bags were displayed separately, 288 images were displayed. In test conditions B and D, 192 images were shown. Even though fewer images were viewed in condition D, compared to conditions A and C, altogether, more time was needed to inspect these test images. While conditions A, B, and C did not differ from each other significantly, large differences were observed between each of these three conditions with condition D (see Table 5). These results indicate that even though fewer images had to be viewed when laptops were kept in passenger bags, altogether more time was needed to apply motion imaging and investigate these images thoroughly. Thus, while motion imaging provides a security advantage, it comes with a certain cost of efficiency.

**Table 5:** **Results of pairwise comparisons with Bonferroni correction for the sums of reaction times (in seconds) of all four conditions (SPSS Bonferroni adjusted *p*-values are quoted)**.

**Comparison**	**Mean difference RT**	***SE***	***p***	***d***
A–B	−58	207.5	1.000	−0.09
A–C	−231	207.5	1.000	−0.32
A–D	−802	207.5	<0.01	−1.14
B–C	−173	207.5	1.000	−0.29
B–D	−744	207.5	<0.01	−1.08
C–D	−570	207.5	<0.05	−0.84

## Summary and conclusions

The benefits of an X-ray machine featuring a new technology offering multiple views of X-ray images and motion imaging were evaluated and compared to single view imaging. In specific, it was investigated whether leaving laptops inside passenger bags resulted in a decrease of detection performance and whether such an effect could be compensated by motion imaging. The results revealed that threat detection performance was much better when laptops and bags were screened separately (see also Mendes et al., 2012). Leaving laptops inside passenger bags resulted in a clear decrease of threat detection performance, supporting the view that increases in superposition and bag complexity affect detection performance negatively (Schwaninger et al., 2005b, 2007; Bolfing et al., 2008). Motion imaging technology could slightly improve threat detection performance. Yet, it could not compensate the negative effect of leaving laptops inside bags. Highest detection performance was achieved when motion imaging was available and laptops and bags were screened separately.

More detailed analyses indicate that performance differed remarkably with regard to the different threat categories [guns, improvised explosive devices (IEDs), knives, others]. IEDs and the others threat category were most difficult to detect, especially when laptops were not removed from passenger bags. Only a small advantage of motion imaging was observed. Merely for the detection of guns, motion imaging seemed to be of substantial benefit. Further analyses regarding the placement of threat items (in bag vs. in laptop) indicated that IEDs were particularly difficult to detect when these were built into the laptop cases. Specifically when laptops were left inside the bags, threat detection performance was quite low compared to when the laptops were displayed separately. Thus, when no automatic explosives detection is available and laptops are not removed from passenger bags, the detection of explosives and bombs, in particular, is impaired. For the categories knives and others, detection performance was higher when these were placed inside the laptops. This could be due to the fact that—for practical reasons—all threat items placed inside the laptops had to be positioned in easy views (canonical views). In general, threat items depicted in more difficult views were harder to detect. These findings are consistent with previous research on viewpoint effects, which showed that recognition of items depicted in frontal/canonical view is easier (e.g., Michel et al., 2007; Bolfing et al., 2008; Koller et al., 2008). Only for the category others, this effect was the other way round. As the category others contained a very heterogeneous group of threat items, possibly screening officers were more familiar with the items positioned in difficult views and thus detected these better. Results also showed that in general more time was needed to inspect the images when laptops were left inside the bags. Longest RTs were found when laptops were not removed from bags and motion imaging was applied. Thus, providing additional views is paid for by increasing RT (see also von Bastian et al., 2008). Even though fewer images were viewed when laptops were left inside the passenger bags, altogether more time was needed to apply motion imaging and inspect these images properly. Keeping factors such as throughput and efficiency at security checkpoints in mind, screening time is an important point to consider.

Technology for security screening will constantly be developed further. Yet, the final decision on whether threat items are contained in luggage still rely on human operators, who inspect the luggage based on an image provided by a machine. The presented study underlines the importance of thoroughly evaluating any new technological features with regard to their added value provided to the screening officers, prior to implementing these in the airport environment. In this study, only a slight benefit of motion imaging technology was revealed. No real advantage could be observed for the detection of IEDs, while the results do suggest that for certain objects such as guns, the rotation and availability of different viewpoints through motion imaging could improve identification. As previous research has shown (e.g., Michel et al., 2007) guns change their shape more drastically than other objects when rotated. Thus, one could assume that motion imaging would possibly be more helpful also for the detection of other threat types if larger rotations and more views are available (or even fully rotatable 3D images, see below).

All in all, the detection of threat items in cabin baggage screening currently still seems more reliable when laptops are taken out of passenger bags. Therefore, the outcomes of this study underline the appropriateness and importance of current regulations specifying that portable computers should be removed from passenger bags for X-ray screening. However, this might be reconsidered if effective and efficient automatic threat detection is available, which is particularly important for IEDs (see e.g., Singh and Singh, 2003; Eilbert, 2009; Mery et al., 2013). Furthermore, if more rotation in depth would be available, higher benefits could possibly be expected, which is of particular importance regarding new technological developments such as computer tomography offering 3D views. Effects of superposition and viewpoint could be reduced further and RTs could be decreased if screening officers can directly navigate to their preferred view of a bag image. In combination with automated threat detection this could possibly result in substantially higher human-machine system performance (see e.g., Flitton et al., 2010; Megherbi et al., 2010). However, this would have to be examined in further studies.

### Conflict of interest statement

The authors declare that the research was conducted in the absence of any commercial or financial relationships that could be construed as a potential conflict of interest.

## Footnotes

^1^For details on the calculation of A′ see section Results and Discussion.

^2^The set was based on a two-month data collection, assessing the contents and types of bags that were passing through the security checkpoints at an international European airport.

^3^Due to the packing condition, the proportion of target present and target absent trials differed for conditions A/C and B/D (in conditions A and C the ratio is 1:2; in conditions B and D the ratio is 1:1). According to signal detection theory (Green and Swets, 1966), different ratios of target present and target absent trials can result in a criterion shift (i.e., changes in hit and false alarm rates). Measures of detection performance in terms of sensitivity such as d' and A' are thought to be relatively independent of criterion shifts, which could also be shown in studies on target prevalence (e.g., Gur et al., 2003; Wolfe et al., 2007; Wolfe and Van Wert, 2010). Therefore, it can be assumed that the different proportions on target present trials in conditions A/C and B/D did not affect detection performance (A′) results in this study.

^4^In all analyses of variance in this study were Mauchly's test indicated that the assumption of sphericity had been violated, the degrees of freedom were corrected using Greenhouse-Geisser estimates of sphericity.

^5^In all *t*-tests of this study where Levene's test indicated unequal variances, degrees of freedom were adjusted using the default procedure in SPSS.
